# Influenza Pandemics of the 20th Century

**DOI:** 10.3201/eid1201.051254

**Published:** 2006-01

**Authors:** Edwin D. Kilbourne

**Affiliations:** * New York Medical College, Valhalla, New York, USA

**Keywords:** Influenza, pandemic, epidemiology, vaccine, recombination, disease, mortality, pneumonia, perspective

## Abstract

Three worldwide (pandemic) outbreaks of influenza occurred in the 20th century: in 1918, 1957, and 1968. The latter 2 were in the era of modern virology and most thoroughly characterized. All 3 have been informally identified by their presumed sites of origin as Spanish, Asian, and Hong Kong influenza, respectively. They are now known to represent 3 different antigenic subtypes of influenza A virus: H1N1, H2N2, and H3N2, respectively. Not classified as true pandemics are 3 notable epidemics: a pseudopandemic in 1947 with low death rates, an epidemic in 1977 that was a pandemic in children, and an abortive epidemic of swine influenza in 1976 that was feared to have pandemic potential. Major influenza epidemics show no predictable periodicity or pattern, and all differ from one another. Evidence suggests that true pandemics with changes in hemagglutinin subtypes arise from genetic reassortment with animal influenza A viruses.

## Abstract 2

Influenza A virus infection created confusion in distinguishing true pandemics, pseudopandemics, and epidemics.

Three worldwide (pandemic) influenza outbreaks occurred in the last century. Each differed from the others with respect to etiologic agents, epidemiology, and disease severity. They did not occur at regular intervals. In the case of the 2 that occurred within the era of modern virology (1957 and 1968), the hemagglutinin (HA) antigen of the causative viruses showed major changes from the corresponding antigens of immediately antecedent strains. The immediate antecedent to the virus of 1918 remains unknown, but that epidemic likely also reflected a major change in the antigens of the virus (*1*)

## Brief Look Back at the 1918 Pandemic

This notorious epidemic is thoroughly and cogently discussed elsewhere in this issue of Emerging Infectious Diseases (*1*). I wish only to add a few points that are not often emphasized, or even mentioned.

The origin of this pandemic has always been disputed and may never be resolved. However, the observations of trained observers at that time are worth noting because they may bear on later genomic analysis of the recently resurrected 1918 virus nucleotide fragments (*1*) and the abortive "swine flu" epidemic of 1976. In Richard Shope's Harvey lecture of 1936 (*2*), he reviews evidence that in the late summer or early autumn of 1918, a disease not previously recognized in swine, and closely resembling influenza in humans, appeared in the American Middle West. Epidemiologic-epizootiologic evidence strongly suggested that the causative virus was moving from humans to swine rather than in the reverse direction. Similar observations were made on the other side of the world and reported in a little-known paper in the National Medical Journal of China (*3*). In the spring of 1918, influenza in humans spread rapidly all over the world and was prevalent from Canton, China, to the most northern parts of Manchuria and from Shanghai to Szechuan. In October 1918, a disease diagnosed as influenza appeared in Russian and Chinese pigs in the area surrounding Harbin. Thus, epidemiologic evidence, fragmentary as it is, appears to favor the spread of virus from humans to swine, in which it remained relatively unchanged until it was recovered more than a decade later by Shope in the first isolation of influenza virus from a mammalian species.

The virus of 1918 was undoubtedly uniquely virulent, although most patients experienced symptoms of typical influenza with a 3- to 5-day fever followed by complete recovery. Nevertheless, although diagnostic virology was not yet available, bacteriology was flourishing and many careful postmortem examinations of patients by academic bacteriologists and pathologists disclosed bacterial pathogens in the lungs (*4*) However, this was a time when bacterial superinfection in other virus diseases could lead to death; for example, measles in military recruits was often fatal (*4*). This information is important in considering the question of "will there ever be another 1918." To the degree that secondary bacterial infection may contribute to influenza death rates, it should at least be partially controllable by antimicrobial agents, as indeed was the case in 1957.

## 1957: Asian Influenza (H2N2)

After the influenza pandemic of 1918, influenza went back to its usual pattern of regional epidemics of lesser virulence in the 1930s, 1940s, and early 1950s. With the first isolation of a virus from humans in 1933 (*5*), speculation began about the possible role of a similar virus in 1918. However, believing that this could have been the case was difficult until the pandemic of 1957. This was the first time the rapid global spread of a modern influenza virus was available for laboratory investigation. With the exception of persons >70 years of age, the public was confronted by a virus with which it had had no experience, and it was shown that the virus alone, without bacterial coinvaders, was lethal (*6*).

### First Recognition of the Pandemic

In 1957, worldwide surveillance for influenza was less extensive than it is today. However, attentive investigators in Melbourne, London, and Washington, DC soon had the virus in their laboratories (*7*) after the initial recognition of a severe epidemic, followed by the publication in The New York Times of an article in 1957 describing an epidemic in Hong Kong that involved 250,000 people in a short period (*8*). Three weeks later, a virus was recovered from the outbreak and sent to Walter Reed Army Institute for Research in Washington, DC for study.

### Nature of the Virus

The virus was quickly recognized as an influenza A virus by complement fixation tests. However, tests defining the HA antigen of the virus showed it to be unlike any previously found in humans. This was also true for the neuraminidase (NA) antigen. The definitive subtype of the Asian virus was later established as H2N2. The new virus had high sialidase/neuraminidase activity, and this activity was more stable than that of earlier strains. Different strains of the Asian virus also differed markedly with respect to sensitivity to either antibody neutralization or nonspecific inhibitors of hemagglutination (*9*). In animal studies, the new H2N2 viruses did not differ in their virulence characteristics from earlier influenza A subtypes. Viral isolates from the lungs of patients with fatal cases showed no discernible differences from those from throat washing isolates of patients without pulmonary involvement within a small circumscribed hospital outbreak (*10*).

### Primary Influenza Virus Pneumonia

Although secondary or concomitant bacterial infections of the lung were found to be a prominent feature of fatal cases in 1918 when a specific etiologic agent was sought (*4*), many cases of rapid death and lung consolidation or pulmonary edema occurred in which bacterial infection could not be demonstrated. As influenza persisted as an endemic disease with regional recurrences after the pandemic, lives continued to be occasionally claimed by abacterial pneumonia.

With the arrival of Asian influenza in 1957, the sheer number of cases associated with pandemicity again brought the phenomenon of primary influenza virus pneumonia to the attention of physicians in teaching hospitals. In contrast to the observations in 1918, underlying chronic disease of the heart or lungs was found in most of these patients, although deaths of previously healthy persons were not uncommon. In the case of carefully studied patients at the New York Hospital, rheumatic heart disease was the most common antecedent factor, and women in the third trimester of pregnancy were among those vulnerable (*11*).

### Response to Vaccination in an Unprimed Population

The pandemic of 1957 provided the first opportunity to observe vaccination response in that large part of the population that had not previously been primed by novel HA and NA antigens not cross-reactive with earlier influenza A virus antigens. As summarized by Meiklejohn (*12*) at an international conference on Asian influenza held 3 years after the 1957 onslaught of H2N2, more vaccine was required to initiate a primary antibody response than with the earlier H1 vaccines (almost always observed in heterovariant primed subjects). In 1958, 1959, and 1960 (as recurrent infections occurred), mean initial antibody levels in the population increased (i.e., subjects were primed) and response to vaccination was more readily demonstrated. Divided doses given at intervals of <4 weeks were more beneficial than a single injection. Less benefit was derived from this strategy as years passed. Intradermal administration of vaccine provided no special advantage over the conventional subcutaneous/intramuscular route, even when the same small dose was given (*13*).

### Nature of Endemic H2N2 Postpandemic Infection

The Asian influenza experience provided the first opportunity to study how the postpandemic infection and disease into an endemic phase subsided. In studies conducted in separate and disparate populations (*14*), the populations compared were Navajo school children and New York City medical students. In both groups, subclinical infections occurred each year during the 3-year study period, and clinically manifested infections decreased in conjunction with an increasing level of H2N2-specific hemagglutination inhibition antibody.

A decreasing incidence of clinically manifested cases can be ascribed either to the increase in antibody levels in the community or to a change in the intrinsic virulence of the virus. Therefore, the nature of the disease during the endemic period is important to define. A study (*15*) in 1960 of hospitalized patients with laboratory-confirmed infections demonstrated a spectrum of disease from uncomplicated 3-day illnesses to fatal pneumonia, all in the absence of discernible epidemic influenza in the community (*15*). Asian (H2N2) virus was destined for short survival in the human population and disappeared only 11 years after its arrival. It was supplanted by the Hong Kong (H3N2) subtype.

## 1968: Hong Kong Influenza (H3N2)

As in 1957, a new influenza pandemic arose in Southeast Asia and acquired the sobriquet Hong Kong influenza on the basis of the site of its emergence to western attention. Once again, the daily press sounded the alarm with a brief report of a large Hong Kong epidemic in the Times of London. A decade after the 1957 pandemic, epidemiologic communication with mainland China was even less efficient than it had been earlier.

As this epidemic progressed, initially throughout Asia, important differences in the pattern of illness and death were noted. In Japan, epidemics were small, scattered, and desultory until the end of 1968. Most striking was the high illness and death rates in the United States following introduction of the virus on the West Coast. This experience stood in contrast with the experience in western Europe, including the United Kingdom, in which increased illness occurred in the absence of increased death rates in 1968–1969 and increased death rates were not seen until the following year of the pandemic.

Since the Hong Kong virus differed from its antecedent Asian virus by its HA antigen, but had retained the same (N2) NA antigen (*16*), researchers speculated that its more sporadic and variable impact in different regions of the world were mediated by differences in prior N2 immunity (*16**–**19*). Therefore, the 1968 pandemic has been aptly characterized as "smoldering" (*19*). Further evidence for the capacity of previous N2 experience to moderate the challenge of the Hong Kong virus was provided by Eickhoff and Meiklejohn (*20*), who showed that vaccination of Air Force cadets with an H2N2 adjuvant vaccine reduced subsequent influenza from verified H3N2 virus infection by 54%.

The amelioration of H3N2 virus infection by NA immunity alone is all the more remarkable because of the capacity of the virus to kill, as occurred in 1918 and 1957, although a broader spectrum of disease severity was apparent in 1968 than in 1957 (*15*). Although not necessarily an indication of virulence, cross-species transmission of the virus was observed (*21*). Thirty-seven years later, the H3N2 subtype still reigns as the major and most troublesome influenza A virus in humans.

## Pseudopandemics and an Abortive Pandemic

### Extreme Intrasubtypic Antigenic Variation and the Pseudopandemic of 1947 (H1N1)

In late 1946, an outbreak of influenza occurred in Japan and Korea in American troops. It spread in 1947 to other military bases in the United States, including Fort Monmouth, New Jersey, where the prototype FM-1 strain was isolated. The epidemic was notable because of the initial difficulty in establishing its cause as an influenza A virus because of its considerable antigenic difference from previous influenza A viruses. Indeed, for a time it was identified as "influenza A prime" (*22*). The 1947 epidemic has been thought of as a mild pandemic because the disease, although globally distributed, caused relatively few deaths. However, as a medical officer at Fort Monmouth, I can personally attest that there was nothing mild about the illness in young recruits in whom signs and symptoms closely matched those of earlier descriptions of influenza (*23*).

Most remarkable was the total failure of vaccine containing a 1943 H1N1 strain (effective in the 1943–1944 and 1944–1945 seasons) to protect the large number of US military personnel who were vaccinated. Previously, antigenic variation had been noted, but never had it been of a sufficient degree to compromise vaccine-induced immunity (*24*). Years later, extensive characterization of HA and NA antigens of the 1943 and 1947 viruses and comparison of their nucleotide and amino acid sequences showed marked differences in the viruses isolated in these 2 years; studies in a mouse model also showed that the 1943 vaccine afforded no protection to the 1947 virus challenge (*24*). Studies in the Fort Monmouth epidemic also documented, by serial bacterial cultures, for the first time the long suspected relationship of influenza to group A streptococcal carriage and disease (*23*).

### 1976: Abortive, Potentially Pandemic, Swine Influenza Virus Epidemic, Fort Dix, New Jersey (H1N1)

In the interest of full disclosure, I predicted the possibility of an imminent pandemic in an op ed piece published in The New York Times on February 13, 1976 (*25*). On February 13, I was notified that influenza viruses isolated from patients at Fort Dix, New Jersey, a few days earlier and provisionally identified as swine influenza viruses were being mailed to my laboratory in New York City. A high-yield (6:2) genetic reassortant virus (X-53) was produced and later used as a vaccine in a clinical trial in 3,000 people. An even higher yielding HA mutant virus, X-53a, was selected from X-53 and subsequently used in the mass vaccination of 43,000,000 people. (I was a member of a Center for Disease Control advisory committee and an ad hoc advisory committee to President Gerald Ford on actions to be taken to protect the American public against swine influenza.) When no cases were found outside Fort Dix in subsequent months and the neurologic complication of Guillain-Barré syndrome occurred in association with administration of swine influenza vaccine, the National Immunization Program was abandoned, and the entire effort was assailed as a fiasco and disaster.

I wish only to note here that my unyielding position on the need for vaccine production and immediate vaccination (not stockpiling) had its basis in what science could be brought to bear in an unprecedented situation. This was the cocirculation in crowded recruit barracks of 2 influenza A viruses of different subtypes: H3N2, the major epidemic virus, and H1(swine) N1. The latter virus, which caused a minor (buried) epidemic and was shown to be serially transmissible in humans, was the putative virus of 1918. Would genetic reassortment of the viruses produce a monster, as is now feared with the current avian virus threat, or did interference by the far more prevalent virus H3N2 suppress further transmission of the swine virus?

Experience had shown a decrease or even disappearance of epidemic viruses in the summer. However, they return in winter to produce disease in conditions favoring transmission: indoor crowding and decreased relative humidity. None of these facts was noted by critics of the program.

### 1977: Russian Flu, a Juvenile, Age-restricted Pandemic, and the Return of Human H1N1 Virus

Our obsession with geographic eponyms for a disease of worldwide distribution is best illustrated by Russian, or later red influenza or red flu, which first came to attention in November 1977, in the Soviet Union. However, it was later reported as having first occurred in northeastern China in May of that year (*26*). It quickly became apparent that this rapidly spreading epidemic was almost entirely restricted to persons <25 years of age and that, in general, the disease was mild, although characterized by typical symptoms of influenza. The age distribution was attributed to the absence of H1N1 viruses in humans after 1957 and the subsequent successive dominance of the H2N2 and then the H3N2 subtypes.

When antigenic and molecular characterization of this virus showed that both the HA and NA antigens were remarkably similar to those of the 1950s, this finding had profound implications. Where had the virus been that it was relatively unchanged after 20 years? If serially (and cryptically) transmitted in humans, antigenic drift should have led to many changes after 2 decades. Reactivation of a long dormant infection was a possibility, but the idea conflicts with all we know of the biology of the virus in which a latent phase has not been found. Had the virus been in a deep freeze? This was a disturbing thought because it implied concealed experimentation with live virus, perhaps in a vaccine. Delayed mutation and consequent evolutionary stasis in an animal host are not unreasonable, but in what host? And if a full-blown epidemic did originate, it would be the first to do so in the history of modern virology, and a situation quite unlike the contemporary situation with H5N1 and its protracted epizootic phase. Thus, the final answer to the 1977 epidemic is not yet known.

## Influenza Pandemics of the 21th Century: the Murky Crystal Ball

All pandemics are different. The minimum requirement seems to be a major change or shift in the HA antigen (1968). In 1957, changes in both HA and NA antigens were associated with higher rates of illness and death. The memorable and probably unique severity of the 1918 pandemic may have depended, at least in part, on wartime conditions and secondary bacterial infections in the absence of antimicrobial drugs. Also, mechanical respirators and supplemental oxygen were not available. Although evidence is strong that recombinational capture of animal influenza HA or NA antigens may be essential for pandemic origins, extreme antigenic drift, such as that which occurred in 1947 (*24*), can lead to global dissemination and disease by the multiply mutated virus.

An intrasubtypic H1N1 animal variant virus (A/H1N1/swine) caused serially transmitted disease, pneumonia, and death at a military installation, yet disappeared within a few weeks (1976, Fort Dix). However, in 1977 an age-restricted pandemic was caused by the revisitation of an H1N1 virus and its ability to infect persons who had not experienced the virus earlier.

Within the brief period of modern virology, of the 16 HA subtypes known to exist, pandemics have been caused only by viruses of the H1, H2, and H3 subtypes. Moreover, serologic and epidemiologic evidence has shown that each of these subtypes has produced pandemics in the past. Are these the default human subtypes? If so, can we be less concerned about the threat of contemporary epizootics?

### Preparing for the Unpredictable

Yes, we can prepare, but with the realization that no amount of hand washing, hand wringing, public education, or gauze masks will do the trick (*27*). The keystone of influenza prevention is vaccination. It is unreasonable to believe that we can count on prophylaxis with antiviral agents to protect a large, vulnerable population for more than a few days at a time, and that is not long enough. How long will they be given? To whom? What are the risks in mass administration? All of this is unknown.

But vaccination against what? We do not know. Perhaps against H5N1. But do we not already have a vaccine? No, we do not; no vaccine of adequate antigenic potency is available in sufficient supply.

The answer lies in an approach first suggested at a World Health Organization meeting in 1969 (*28*) and repeatedly endorsed since by virtually every pandemic preparedness planning group. This recommendation assumes that the nature of the next pandemic virus cannot be predicted, but that it will arise from 1 of the 16 known HA subtypes in avian or mammalian species. Accordingly, preparation by genetic reassortment of high-yield seed viruses of all HA subtypes should proceed as soon as possible for potential use in vaccine production (*28*). Thirty-seven years later, this goal has not yet been achieved. Reassortant viruses have been used in vaccine production since 1971 in response to the emergence of antigenic drift variants. A repository at the National Institute of Allergy and Infectious Diseases (http://www.flu-archive.org) contains recently made early and late H2N2 candidate vaccine reassortant viruses that could address the return of that virus subtype, a high-yield H7N7 reassortant virus, and a high-yield H5N3 wt mutant that does not kill either chickens or fertile hen eggs (E.D. Kilbourne, M. Perdue, unpub. data). Recently, a high-growth vaccine strain has also been developed as a pandemic vaccine candidate for protection against the threat of H9N2 virus (*29*) by what has become the standard technique of reassortment with A/PR/8/34 (H1N1) virus (*28*).

One concern about previous and anticipatory preparation and characterization of high-yield reassortants is that they may not exactly match the newly emerging strain of that subtype. Perhaps not, but in the face of a pandemic threat they could serve as barricade vaccines (*27*), ready to be pulled out of the freezer at the first threat from any subtype.

## Postscript

### Back to Reality: Urgent Questions That Can and Should be Answered Immediately

In assessing pandemic risk, we seem to have forgotten that influenza virus contains not 1, but 2 immunogenic protective antigens. As a case in point, I am not satisfied that we have sufficiently examined immunity to the N1 antigen of the H5N1 pandemic-candidate virus. Did infected persons who died lack antibody to N1 in their acute-phase sera? To what extent, if any, do the N1 antigens of human strains crossreact with those of the H5N1 variants? Is the antibody response to N1 antigen being examined in recipients in recent H5N1 virus vaccine trials? Mindful of the damping effect of N2 antibody in the 1968 pandemic, we might find reassurance and explanations in learning these results.

Dr Kilbourne, emeritus professor of microbiology and immunology at New York Medical College, has spent his professional life in the study of infectious diseases, particularly virus infections. His early studies of coxsackieviruses and herpes simplex preceded study of influenza in all of its manifestations. Primary contributions have been to understanding of influenza virus structure, genetics, molecular epidemiology, and pathogenesis. His studies of influenza virus genetics resulted in the first genetically engineered vaccine for the prevention of human disease, and a new approach to influenza immunization received 2 US patents.
